# The role of electrolyte imbalances in predicting the severity of COVID-19 in the hospitalized patients: a cross-sectional study

**DOI:** 10.1038/s41598-022-19264-8

**Published:** 2022-08-30

**Authors:** Fatemeh Yasari, Meshkat Akbarian, Atefeh Abedini, Maryam Vasheghani

**Affiliations:** 1 Chronic Respiratory Diseases Research Center, National Research Institute of Tuberculosis and Lung Diseases (NRITLD), Shahid Beheshti University of Medical Sciences, Masih Daneshvari Hospital, Darabad Avenue, Shahid Bahonar Roundabout, Tehran, 1956944413 Iran; 2 Internal Medicine Department, Medical School, Shahid Beheshti University of Medical Sciences, Tehran, Iran

**Keywords:** Biochemistry, Physiology, Diseases, Health care, Medical research, Nephrology, Risk factors, Signs and symptoms

## Abstract

Coronavirus disease 2019 (COVID-19) can be fatal in severe cases. Accordingly, predicting the severity and prognosis of the disease is valuable. This study examined the role of electrolyte imbalances in predicting the severity of COVID-19. In this cross-sectional study, 169 hospitalized patients with COVID-19 were included and categorized into three groups based on the severity of the disease (moderate, severe, and critical). Serum levels of electrolytes (calcium [Ca], phosphorus [P], sodium [Na], potassium [k], and magnesium [Mg]), inflammatory markers (D-dimer, C-reactive protein [CRP], ferritin, and lactate dehydrogenase [LDH]), and 25OHVitamin D were measured. The mean age of patients was 53 years, and 54% were male. They had moderate, severe, and critical illnesses in 22%, 47%, and 31%, respectively. CRP, D-dimer, and ferritin increased with the severity of the disease. The lower median values of Mg, Na, 25OHVitamin D, Ca, LDH, and higher median lymphocyte counts were observed in the moderate vs. the severe group (*P* < 0.05). These parameters have acceptable sensitivity and specificity at the suggested cut-off level to discriminate the moderate and critical cases. Serum parameters introduced in this study are appropriate for differentiating between critical and moderate cases. The electrolyte imbalance can predict critical patients.

## Introduction

In late 2019, a new pandemic emerged from the severe acute respiratory syndrome coronavirus 2 (SARS-CoV-2), which the World Health Organization called coronavirus disease 2019 (COVID-19)^1^. The manifestations of COVID-19 are highly variable from mild^2^ to severe pneumonia, acute respiratory distress syndrome death^3^. SARS-COV-2 enters the host cells via angiotensin-converting enzymes 2 (ACE2) receptors, expressed in several organs, such as the heart, liver, kidneys, and lungs with several physiological roles, including regulation of the renin-angiotensin system, blood pressure, and electrolytes balance^4^.

The four essential serum electrolytes are sodium, potassium, calcium, and magnesium. The association of hyponatremia with pneumonia has been proposed for many years. Hyponatremia in people with community-acquired pneumonia has been associated with an increased risk of admission to the intensive care unit (ICU), hospital length of stay, and mortality^5^. In a study on patients with COVID-19 in India, 44% of them had hyponatremia, which was mostly mild. In these patients, hyponatremia has been associated with the increased rate of ICU admission and mortality^6^. In patients with COVID-19, hypokalemia is a frequent laboratory abnormality. Hypokalemia may increase the risk of arrhythmia. Hypokalemia was diagnosed in (41%) hospitalized patients. The average serum potassium was 3 meq/L. Hypokalemia was mild in the most of patients and was associated with hypocalcemia in the half of them. Female gender and diuretic therapy were identified as risk factors for low serum potassium levels. Hypokalemia was not associated with ICU admission and death in this group of patients^7^. In another study from China, it has been shown that hypokalemia has a high prevalence in patients with COVID-19, and there is a direct relationship between the degree of hypokalemia and the severity of COVID-19^8^. Hypocalcemia is common in severe COVID-19 and is associated with worsening prognosis and outcome^9^. The results of a meta-analysis on 12 articles with a population of 2891 patients showed that 59% of people with COVID-19 have hypocalcemia. Hypocalcemia was significantly associated with the severity and mortality of disease, length of hospital stays, and admission to the ICU in patients with COVID-19^10^. In a study, hypermagnesemia is one of the signs of disease severity in hospitalized patients with COVID-19, and it is recommended that serum magnesium should be added to the panel of routine tests on COVID-19^11^. Hypophosphatemia was seen in 7.6% of patients at admission to the hospital. In people with hypophosphatemia, the risk of respiratory failure and mortality was significantly higher. The survival rate of the hypophosphatemia group was significantly lower than the normophosphatemia group^12^. Patients with lower vitamin D levels show an increased risk of ICU admission or death from SARS-CoV-2 infection^13,14^.

The electrolyte disturbances are caused by the direct effect of the virus on infected host cells. Furthermore, the malfunction of the organs during the disease can also cause electrolyte imbalance^7^. Possible causes such as fever, hyperventilation, sweating, drug-related side effects, and dietary changes may cause electrolyte imbalances in patients with COVID-19^5^.

The electrolyte imbalances (such as hypokalemia, hyponatremia, and hypocalcemia) are more common in patients with severe COVID-19 in some studies^15^. However, some other studies have not confirmed these associations^16,17^. There is controversy in the results of studies and lack of evidence on the diagnostic accuracy and optimal cut-off of each serum parameter for estimation of the disease severity. The global rate of infection and death due to this pandemic is still on the rise, and more people are infected by the coronavirus^18^. The electrolytes are routinely assessed for the patients with COVID-19. Their possible role in the prediction of the severity of COVID-19 can suggest them as easy and accessible predictors. Therefore, research continues on different aspects of this pandemic. Considering the prevalence and effects of electrolyte imbalance on the severity of COVID-19 these factors and electrolytes were investigated in this study. The present study aimed to investigate the predictive value of electrolytes imbalance and inflammatory markers in the hospitalized patients with COVID-19, according to their disease severity.

## Materials and methods

### Study design

In this cross-sectional study, patients who were referred to Masih Daneshvari Hospital, Tehran, Iran, from March 6^th^ to the end of June 2020, and had been diagnosed with COVID-19, based on PCR results, were included.

Age is one of the most critical risk factors for the severity of COVID-19 and its mortality^19^. The findings of studies conducted in China and Italy show that age, cardiovascular disease, high blood pressure, and diabetes mellitus increase the risk of death among affected patients. Age, male gender and multiple co-morbidities definitely increased the mortality rate in the hospitalized patients with COVID-19 from Italy^20,21^. Chronic kidney and liver disease and the use of drugs such as diuretics can affect the blood levels of electrolytes and are considered confounding variables^22^. Drugs such as angiotensin-converting enzyme (ACE) inhibitors may play a role in the pathophysiology of COVID-19 by altering electrolyte or ACE levels^23^. Therefore, we excluded conditions and diseases that contributed to the severity of COVID-19 and its mortality or change in the serum level of electrolytes^19,22–24^. We also excluded the people taking above mentioned drugs. *Exclusion criteria:* Elderly patients (over 75 years), patients with end-stage renal disease, hepatic failure, and acute cardiovascular problems (such as myocardial infarction), patients with recent use of diuretics, ACE inhibitors, beta-blockers, non-steroidal anti-inflammatory drugs, and calcium or magnesium supplements.

The sample size was calculated at 167, considering *P* = 70.4% and q = 29.6%, based on the study by Wan et al.^25^, the significance level of 0.05, and power 80%. The researcher enrolled the eligible patients (according to the inclusion criteria stated above) using the census method; before enrollment, the researcher explained the research objectives to the patients and asked them to read and sign the written informed consent sheets.

To collect information, the field method was used and the study variables were recorded into the study checklist by reviewing patients’ records and interviewing the patients. The collected information included demographics (age and gender) and the information needed to classify patients based on disease severity, including clinical symptoms (such as shortness of breath, fever, cough, loss of consciousness, gastrointestinal symptoms), computed tomography (CT) scan findings, respiratory rate (RR) per minute, oxygen saturation (SpO_2_) at rest, mean arterial blood oxygen pressure to respiratory oxygen concentration ratio (PaO_2_/FiO_2_), respiratory failure requiring mechanical ventilation, admission to ICU, and shock. Accordingly, the severity of the disease was categorized into four classes: mild (mild clinical symptoms and no imaging), moderate (fever and/or respiratory symptoms, or imaging evidence of pneumonia), severe (RR ≥ 30 per minute, SpO_2_ ≤ 93%, or PaO_2_/FiO_2_ ≤ 300 mmHg), and critical (respiratory failure requiring mechanical ventilation, shock, multiple organ dysfunction or need for monitoring and treatment in the ICU)^26^. Mild cases did not require hospitalization and were not included in the present study.

One venous blood sample was taken from the brachial vein of all patients at admission and sent to the laboratory for measurement of lymphocyte count, C-reactive protein (CRP), serum levels of 25OHVitamin D, ferritin, D-dimer, and electrolytes, including sodium (Na), calcium (Ca), phosphorus (P), potassium (K), and magnesium (Mg). The EDTA-containing tubes were used for lymphocyte count, measured using cell counter Sysmex KX-21 (with a normal range of 1360–3740) and serum was used for the rest of serum parameters. CRP, Na, K, Ca, Mg, P, and LDH were measured using Chemistry Analyzer Spectrophotometry BT3000; the normal range of CRP is < 10 mg/L in adults, Na is 135–145 meq/L, K is 3.5–5.3 meq/L, Ca is 8.6–10.3 mg/dL, Mg is 1.5–2.6 mg/dL, P is 2.5–5 mg/dL in adults, and LDH is 225–500 U. 25OHVitamin D was measured using Chemiluminescence Abbott 1000 SR and values < 10 ng/mL were considered as a severe deficiency, 10–19.9 ng/mL as moderate deficiency, 20–29.9 ng/mL as insufficiency, 30–100 ng/mL as sufficient, and > 100 ng/mL as high^14^. Citrated tubes were used for the measurement of D-dimer and ferritin. D-dimer was measured using min UIDAS, Enzyme-linked fluorescent assay; values ≥ 500 ng/mL were considered as positive. Ferritin was measured using Chemiluminescence Abbott 1000 SR; the normal range of ferritin was 21–275 ng/mL in men and 4.6–204 ng/mL in women.

### Statistical analysis

Data analysis was performed using SPSS software version 22 (IBM Corp. 2013. Armonk, NY: IBM Corp.). After data was input into the software, to describe the data, the number and percentage were used for qualitative variables. The normality of numeric variables was checked using the Shapiro–Wilk test. Since none of the numerical data had a normal distribution, the median was used to describe the data and Kruskal–Wallis statistical test was used to compare between the three groups and the posthoc test was used for pairwise comparisons. Only two of the variables had normal distribution (potassium and phosphorus), reported as mean ± standard deviation (SD) and compared among the three groups using One-Way ANOVA. In the next step, we calculated the diagnostic accuracy of the parameters, which showed a significant difference between moderate and critical patients using Med Calc software version 20.013 (MedCalc Software Ltd, Ostend, Belgium; 2021). ROC diagram was drawn and sensitivity, specificity, and under the curve (AUC) were reported with optimal cut-off. In all tests, a significance level of < 0.05 was considered.

### Ethics

The proposal of this study was approved by Committee for ethics in biomedical research of the National Research Institute of Tuberculosis and Lung Diseases (NRITLD), Shahid Beheshti University of Medical Sciences with ID: IR.SBMU.NRITLD.REC.1399.205 on January 11, 2021. All stages of the study were conducted according to the guidelines of this committee.

## Results

A total of 169 patients completed the study; 91 were men (53.8%). The median age of the participants was 55 years. Thirty-seven patients had the moderate disease (21.9%), 80 patients had severe disease (47.3%), and 52 patients had the critical disease (30.8%); the sex distribution of the three groups was not different (*P* = 0.129).

The total values of the serum parameters and their comparison among the three groups are shown in Table 1. As shown in the table, the median age of patients was different among the three groups (*P* = 0.003) and higher in critical vs. moderate and severe vs. moderate (*P* = 0.009 and 0.011, respectively). As presented in Table 1, CRP, D-dimer, and ferritin showed a severity-dependent trend and increased with the increase in the disease severity (*P* < 0.05; Table 1). But the results showed no difference in serum levels of potassium and phosphorus among the three groups with different disease severities (*P* > 0.05; Table 1). The rest of the serum parameters were different between some and not between other groups: median lymphocyte count was higher and median Mg level was lower in moderate than critical patients (*P* = 0.001 and < 0.001, respectively). Serum levels of Na were lower and 25OHVitamin D was higher in the moderate and severe groups vs. critical group (*P* < 0.05; Table 1). Median Ca and LDH levels were lower in the moderate group, compared to severe and critical groups (*P* = 0.032 and 0.021, respectively).

**Table 1:** Comparison of age and the serum parameters among the three study groups.

	Total	Moderate disease (N = 37)	Severe disease (N = 80)	Critical disease (N = 52)	*P* value*	*P* value^†^	*P* value^‡^	*P* value^§^
Age	55 (42–62)	47.00	57.00	58.00	0.003	0.011	1.00	0.009
Lymphocyte	1453 (1020.25–1941)	1677.00	1410.00	1090.00	0.001	0.490	0.524	0.001
CRP	30 (13–48)	5.00	25.00	47.50	< 0.001	< 0.001	< 0.001	< 0.001
Sodium	139 (136–140)	140.00	139.00	135.50	< 0.001	0.872	< 0.001	< 0.001
Potassium	4.07 ± 0.54	3.96 ± 0.26	3.99 ± 0.48	4.26 ± 0.72	0.347	–	–	–
Calcium	8.80 (8.40–9.50)	9.00	8.80	8.55	0.038	0.021	0.847	0.032
Phosphorus	3.11 ± 1.11	3.26 ± 0.76	3.04 ± 0.85	3.11 ± 1.57	0.457	–	–	–
Magnesium	2.20 (1.90–2.50)	2.40	2.20	1.90	< 0.001	0.490	0.524	< 0.001
25OHVitamin D	20 (13–27)	23.5	21	14	0.014	0.606	0.042	0.015
D-dimer	702.5 (300–2402.5)	300	600	3120	< 0.001	< 0.001	< 0.001	0.008
Ferritin	441 (200–1000)	150	439	1000	< 0.001	< 0.001	< 0.001	0.001
LDH	518 (413.75–618.75)	394.5	519.00	660.00	< 0.001	0.004	0.141	< 0.001

*CRP* C-reactive protein; *LDH* lactate dehydrogenase, all values are reported as median, except potassium and phosphorus, reported as mean ± standard deviation.

*Comparison among the three groups using Kruskal Wallis test, ^†^Comparing moderate with severe, ^‡^Comparing severe with critical, ^§^Comparing moderate with critical.

**Figure 1:**
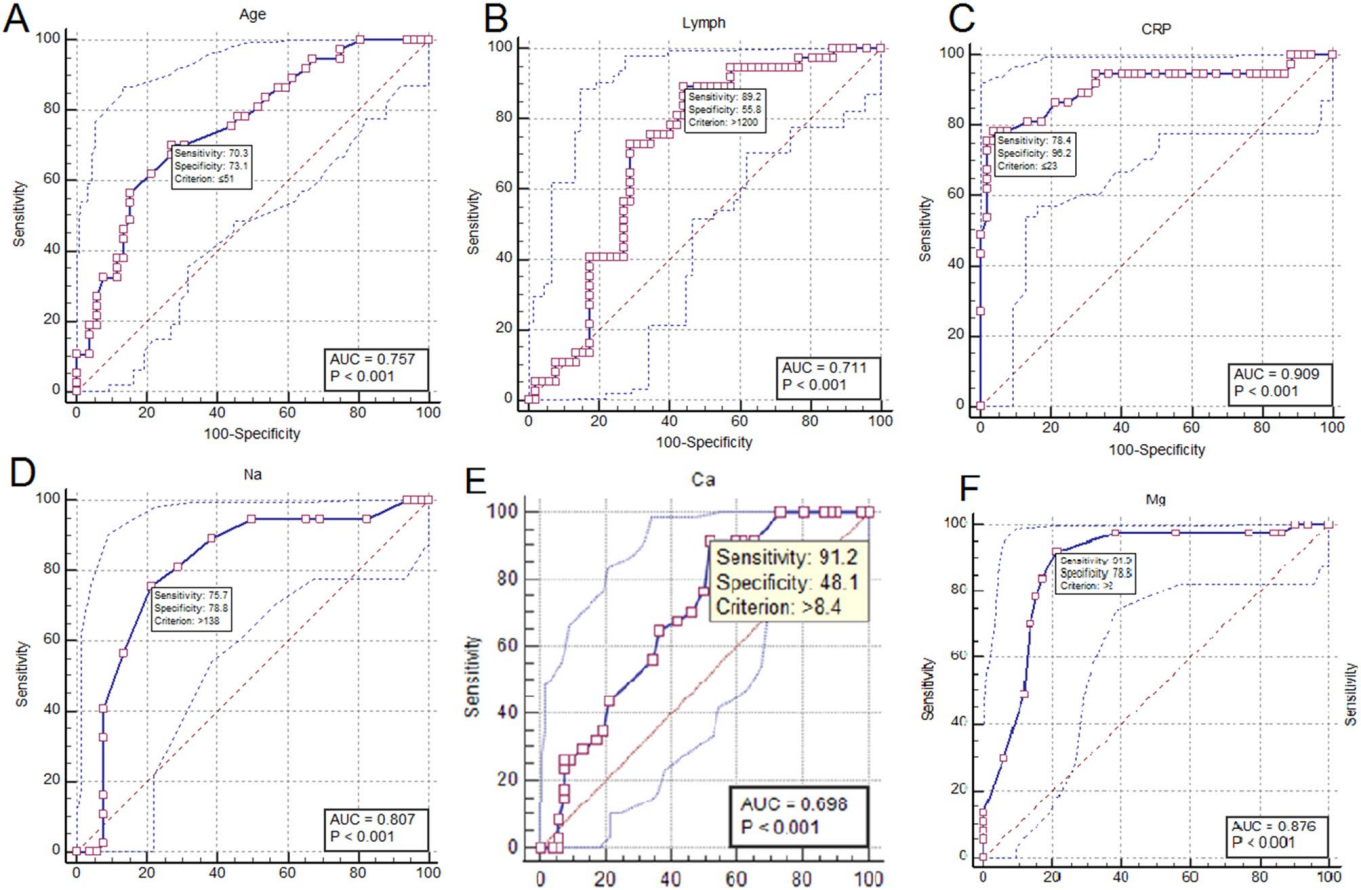
The diagnostic accuracy of age and serum parameters for discrimination of critical cases from moderate.

**Figure 2:**
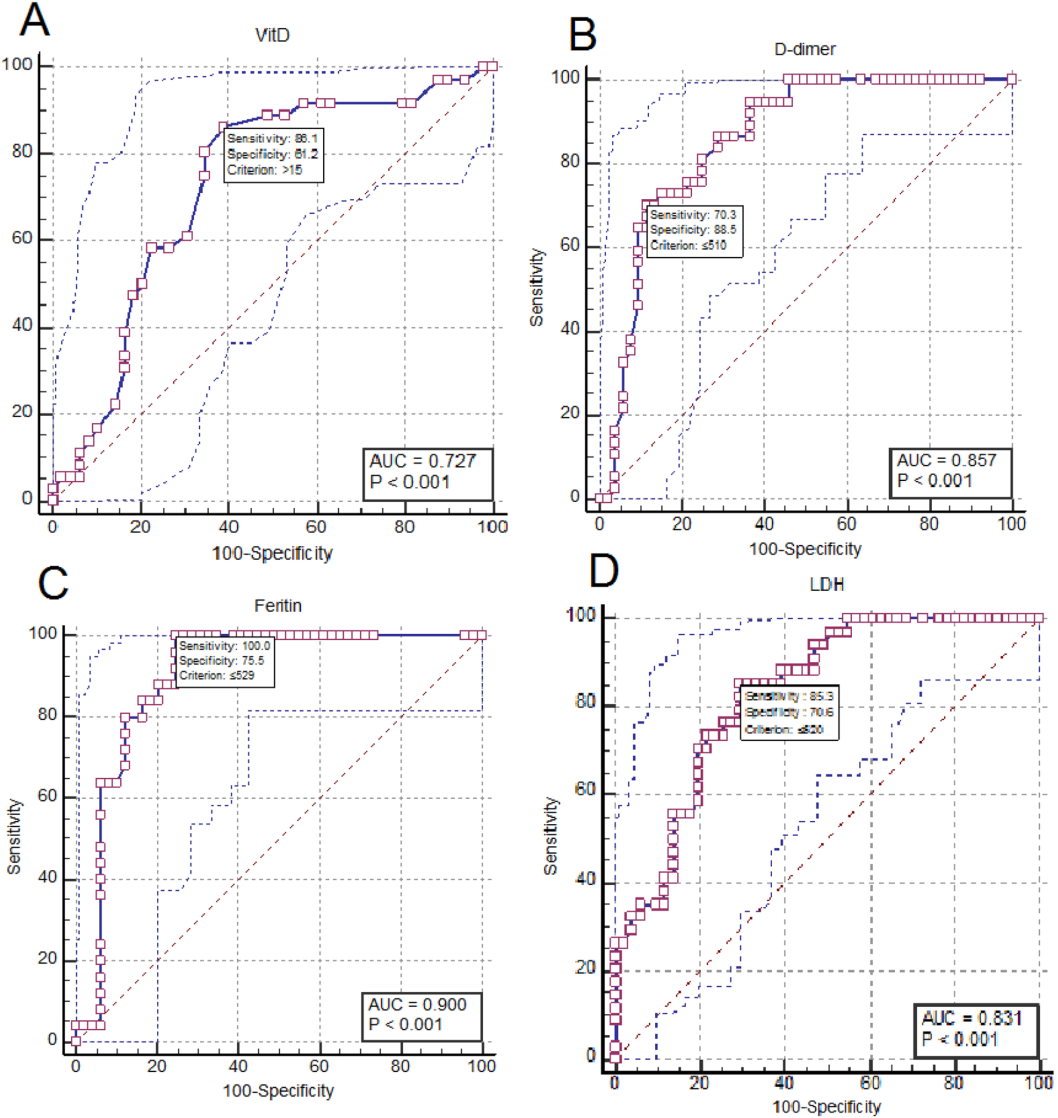
The diagnostic accuracy of 25OHVitamin D, D-dimer, ferritin, and LDH for discrimination of critical cases from moderate.

Investigating the diagnostic accuracy of the parameters with a significant difference between moderate and critical patients showed the sensitivity, specificity, and area under curve (AUC) were reported with optimal cut-off of the serum parameters. As shown in Fig. 1, sensitivity and specifity of age ≥ 51 years were 70.3% and 73.1% (Fig. 1A), lymphocyte count ≤ 1200 cells/mm^3^ were 89.2% and 55.8% (Fig. 1B), CRP ≥ 23 mg/L were 78.4% and 96.2% (Fig. 1C), Na ≤ 138 meq/L were 75.5% and 78.8% (Fig. 1D), Ca ≤ 8.4 mg/dL were 91.2% and 48.1% (Fig. 1E), Mg ≤ 2 mg/dL were 91.9% and 78.8% (Fig. 1F) respectively, for discrimination of critical from moderate cases. The sensitivity and specificity of 25OHVitamin D ≤ 15 mg/dL were 88.1% and 61.2% (Fig. 2A), D-dimer ≥ 510 µg/mL were 70.3% and 88.5% (Fig. 2B), ferritin ≥ 529 ng/mL were 100% and 75.5% (Fig. 2C), and LDH values ≥ 520 U/L were 85.3% and 70.6% (Fig. 2D), respectively, for discrimination of critical from moderate cases. The AUCs are shown on each figure.

## Discussion

The comparison of the serum parameters among the patients with the different severity of COVID-19 (moderate, severe, and critical) showed that the most serum parameters (including the electrolytes and inflammatory markers; except K and P) were different among the three groups. Of note, CRP, D-dimer, and ferritin showed a severity-dependent trend and increased with the increase in the disease severity and lower median values of Mg, Na, 25OHVitamin D, Ca, and LDH and higher median lymphocyte count were observed in moderate group vs. severe group. Previous studies on COVID-19 have addressed some (but not all) of these parameters, as discussed below.

The results of the present study showed a high sensitivity for the lymphopenia as an important predictor of the critical COVID-19. In a retrospective study, the lymphocyte count of the patients admitted to ICU was significantly lower and these patients had a higher risk of acute kidney injury^27^. Although the above-mentioned studies have not calculated the diagnostic accuracy of this parameter, their results are consistent with that of the present study.

Three of the four inflammatory markers, evaluated in the current study, CRP, D-dimer, and ferritin, showed a severity-dependent increasing trend and all had high sensitivity and specificity for discrimination of critical from moderate cases. Several mechanisms recommended to play a role in the inflammatory and coagulation dysregulation during the COVID-19 course can be the underlying mechanism for this finding^28^. In a study on patients with COVID-19, CRP with a cut-off level of 11 mg/dL was suggested to have a sensitivity of 72% and a specificity of 71% to distinguish severe from moderate ARDS in COVID-19 patients^29^, which is almost consistent with the results of present study. Similar results were obtained for D-dimer, ferritin and LDH.

Of note, ferritin showed 100% sensitivity in the present study, which show its association with disease severity, death, and thrombo-embolic events. This finding is in line with the results of previous studies and suggest ferritin as an available and cost-effective method for the prediction of COVID-19 prognosis^30–32^. LDH also showed a high sensitivity and specificity at cut-off level of 520 U/L. This finding is similar to the results of previous studies, although the suggested cut-off levels and diagnostic accuracy rate are different^29,33^.

Three of the five electrolytes evaluated in the present study, Na, Mg, and Ca, showed high diagnostic accuracy for the prediction of critical COVID-19.

The suggested cut-off level of sodium for predicting critical COVID-19 was 138 meq/L in this study. This cut-off level is within the normal limits of sodium serum level (135–145 meq/L); therefore, we do not propose its use as a predictor. But hyponatremia is associated with the severity of disease in this study. This is in line with the results of previous studies which showed higher rates of hyponatremia in patients with severe COVID-19^15,25,34^ and its predictive value for death, ICU admission, and need for intubation^35–37^. However, none of these studies initially included critically ill patients with COVID-19. Sodium plays an important role in ACE2 expression and electrolyte imbalance in COVID-19. Also, the level of sodium decreases in the more severe disease^38^. A multicenter study including European and Latin American countries has investigated the relationship between hyponatremia (Na < 135 meq/L) and hypernatremia (Na > 145 meq/L) with COVID-19 on 4664 hospitalized patients. In this study, the prevalence of hyponatremia and hypernatremia was 20.5% and 3.7% respectively. Sodium imbalances were associated with increased risk of sepsis and mortality^36^. The underlying mechanism for the hyponatremia in COVID-19 is supposed to be related to low intake due to anorexia, excretion of sodium from skin through sweating, loss of sodium from the gastrointestinal system due to vomiting or diarrhea, increased excretion of sodium from kidney due to the use of diuretic drugs or decreased excretion of water from kidney due to the syndrome of inappropriate secretion of antidiuretic hormone (SIADH). This syndrome occurs as a result of increased release of inflammatory cytokines^7,37,39^. Previous studies have suggested the role of sodium serum levels in regulating and improving immune system function. Hypernatremia in the elderly with COVID-19 has increased mortality. Hyponatremia can aggravate COVID-19 or be a sign of severe illness. Therefore, sodium disorders in both spectra can affect the severity and mortality of this disease^40^.

In this study, the mean serum potassium level increased with the disease severity, but this difference between the groups was not statistically significant. Patients with COVID-19 have had hypo- and hyper-kalemia in 24% and 4% of cases, respectively. The ratio of urinary potassium to creatinine was measured in 39% of patients and 95.5% of them had increased urinary potassium excretion. Hypokalemia in the setting of COVID-19 can increase the risk of ARDS, arrythmia, and acute heart injury. Hypokalemia in the setting of SARS-CoV2 infection may result from overactivation of the renin–angiotensin–aldosterone system (RAAS), low intake, and gastrointestinal or kidney loss. According to the previous studies, the virus increases angiotensin II level and decreases potassium level by binding to ACE2 and reducing its expression^38^. Anorexia secondary to sever acute illness or comorbidities, gastrointestinal loss due to diarrhea, and kidney loss due to diuretic use or tubular injury from ischemia or nephrotoxic toxins are important pathophysiology of hypokalemia in COVID-19. According to this latter hypothesis, tubular damage may be related to the direct cytotoxic effect of SARS-CoV-2, as the virus is associated with diffuse tubular damage^7^. There is high heterogeneity in different studies in terms of the prevalence and severity of the potassium disturbances. Therefore, the role of potassium disturbances in the COVID-19 and its complication is unclear, so further studies are needed^41^.

Magnesium, an essential element for basic biochemical reactions, participates in a variety of physiological functions and normal metabolism, such as potassium ion or calcium ion transport, energy metabolism, protein and nucleic acid synthesis. Magnesium also reduces inflammation and is a muscle relaxant, vasodilator, and has antioxidant effects and protects the nervous system. Therefore, Magnesium homeostasis affects the health of the reproductive, cardiovascular, nervous, respiratory, and digestive systems. In the review of possible causes of hypomagnesemia in COVID-19, obesity, type 2 diabetes, high arterial blood pressure, decreased immune response, and cytokine storm are mentioned. People over the age of 65 and people with underlying diseases such as obesity, type 2 diabetes (T2DM) are more at risk of poor outcomes from COVID-19. Hyperinsulinemia is common in all of the above cases. Hyperinsulinemia causes hypomagnesemia through increased renal excretion of Magnesium and the intracellular level of this ion decreases. Hyperinsulinemia causes a decrease in vitamin D levels through hypomagnesemia, a decrease in vitamin D absorption, vitamin D trapping in fat tissue, or a decrease in vitamin D activation capacity. These changes decrease the activity of sulfotransferase enzyme 2B1b (SULT2B1b), as a result of which the sulfurylation of cholesterol to cholesterol sulfate is reduced. The reduction of cholesterol sulfate reduces the electrical charge of red blood cells and increases the risk of agglutination and thrombosis^42,43^. The low serum level of Magnesium and Na has been shown in patients who are admitted to ICU in a study from Iran^44^. Special attention has been paid to Magnesium in COVID-19, as there seems to be a bi-directional relationship between hypomagnesaemia and COVID-19 prognosis; COVID-19 impairs Magnesium homeostasis and Magnesium deficiency increases the sensitivity of endothelial cells to oxidative stress and the resulting endothelial dysfunction causes decreased fibrinolysis, which increases the risk of coagulation^45^. Magnesium also plays a role in the immune response through regulating the cytotoxicity of natural killer cells and CD8 killer T cells^46^.

In the present study, three parameters (Mg, Ca, and 25OHVitamin D) were shown as significant predictors of critical COVID-19. Magnesium is important for activating vitamin D, which plays a protective role against oxidative stress. Vitamin D and Magnesium supplementation in the patients with COVID-19 has reduced the severity of disease and its complications^42,47^. Previous studies have also found the association of vitamin D deficiency with poor outcomes, such as need to mechanical ventilation and death^48^. The mechanism of the association between vitamin D deficiency and COVID-19 severity can be related to the role of vitamin D on immune system. Vitamin D balances the immune system in such a way that it strengthens the immune system against the virus but prevents the cytokine storm by T regulatory lymphocytes (Tregs). These especial immune cells are the main defense against uncontrolled inflammation and viral infection, which reduce in patients with severe COVID-19 and vitamin D deficiency^49^.

Calcium is one of the four essential ions of the body whose regulation is related to Magnesium and vitamin D. In this study the hypocalcemia was associated with the severe and critical disease. This finding is in line with the results of previous studies^50,51^. The cut-off point of calcium level with appropriate accuracy, which was determined to predict COVID-19 severity in this study, is in normal range. Therefore, we do not recommend using this parameter for prediction of disease severity. Hypocalcemia occurs in patients with COVID-19 by several mechanisms, such as anorexia and malnutrition during acute illness, change in blood levels of unsaturated fatty acids due to the inflammatory process, hypomagnesemia, and vitamin D deficiency. Recent studies have suggested other mechanisms, including decreased intestinal absorption of calcium and parathyroid dysfunction. Calcium absorption from the small intestine is further reduced by chronic vitamin D deficiency during severe COVID-19 infection. Parathyroid dysfunction is more common in this condition, and only one-fifth of these patients have an adequate parathyroid response to hypocalcemia. In acute respiratory failure due to viral infections, researchers have been able to find RNA virus and antigenic substances in parathyroid cells. The ACE 2 receptor has also been detected in these cells. In the acute critically ill patients with COVID-19, as in other acute systemic diseases, parathyroid function decreases with increasing inflammatory response and cytokine levels, and hypocalcemia intensifies. This type of parathyroid hypofunction is more prominent in men^52^.

In the context of SARS-CoV-2 infection, there may be a cytokine storm that consumes ATP, and phosphate and magnesium are needed to reproduce ATP. In fact, the decrease of phosphorus is seen more in patients who have conditions such as old age, diabetes, obesity, and the use of diuretics^53^. Decreasing the level of phosphorus at the same time as the SARS-CoV-2 enters the body increase the risk of infection. But due to hypophosphatemia, immune responses are weakened and as a result cannot repair the damage to cells and tissues, which leads to the progression of the disease^36^.

One of the limitations of the present study was the nonrandomized recruitment of participants from single center, that may affect the results. Therefore, the extrapolation of the results to the whole population should be performed with great caution. Furthermore, we did not follow the patients and could not report the short- or long-term outcomes. Another limitation of the present study is its cross-sectional nature. If a longitudinal study is designed and serial blood sampling are taking from patients, it is better to comment on the changes of these parameters in the course of the disease and outcomes.

## Conclusion

In conclusion, the results of the present study showed the diagnostic accuracy of several serum parameters for the prediction of critical cases of COVID-19. As these serum parameters are often measured in patients with COVID-19 in routine patient work-up, their measurement does not require additional costs, equipment, or time; therefore, we recommend that physicians can use these parameters as easy, accessible, and cost-effective markers for predicting COVID-19 severity at the proposed cut-offs. Earlier prediction of critical cases, may be reduce mortality by appropriate therapeutic and preventive measures. Future longitudinal randomized clinical trials with larger sample size, can better determine the relationship between electrolyte imbalances with the severity of COVID-19 and its outcomes.

## Data Availability

All authors had online access to all data during the study. All the data obtained during this study are published in this article. According to the provisions of the written consent of the patients, it is stipulated that the data of the study will be published without mentioning the names of the participants and in the form of collective data.
